# Long-term cognitive impairment after ICU treatment: a prospective longitudinal cohort study (Cog-I-CU)

**DOI:** 10.1038/s41598-020-72109-0

**Published:** 2020-09-23

**Authors:** Annekatrin Müller, Judith von Hofen-Hohloch, Meinhard Mende, Dorothee Saur, Christopher Fricke, Sven Bercker, Sirak Petros, Joseph Classen

**Affiliations:** 1 Department of Neurology, University of Leipzig Medical Center, Liebigstr. 20, 04103 Leipzig, Germany; 2 Institute for Medical Informatics, Statistics and Epidemiology, University of Leipzig, Leipzig, Germany; 3 Department of Anaesthesiology and Intensive Care Medicine, University of Leipzig Medical Center, Leipzig, Germany; 4 Medical Intensive Care Unit, University of Leipzig Medical Center, Leipzig, Germany

**Keywords:** Medical research, Risk factors

## Abstract

In this prospective cohort study we aimed to investigate the trajectory of the cognitive performance of patients after discharge from an intensive care unit (ICU). Special consideration was given to patients with suspected premorbid cognitive impairment who might be at risk for the development of dementia. Clinical characteristics were collected until discharge. The premorbid cognitive state was estimated by a structured interview with a close relative. Cognitive outcome was assessed using the Consortium to Establish a Registry of Alzheimer’s Disease (CERAD) Plus battery and the Stroop Color and Word Test at the time of discharge from ICU and 9 months later. The results of the study group were compared to an established healthy control group and to normative data. A total number of 108 patients were finally included. At the time of discharge, patients underperformed the healthy control group. In linear regression models, delirium during the ICU stay and the factor premorbid cognitive impairment were associated with poorer cognitive outcome (p = 0.047 and p = 0.001). After 9 months, in 6% of patients without evidence of premorbid cognitive impairment long-lasting deficits were found. In patients with suspected premorbid cognitive impairment, performance in tests of executive function failed to improve.

## Introduction

Cognitive deficits after treatment in an intensive care unit (ICU) have long been considered an epiphenomenon that resolves with recovery from critical illness. However, there is now growing evidence that cognitive impairment after critical illness may reflect a longer lasting encephalopathy^1–5^ which is independent from the primary disorder. The mechanisms leading to cognitive impairment after critical illness are poorly understood. It is largely unknown whether or not medical treatment or in-hospital complications contribute to its appearance. Among several factors, delirium has the strongest association with post-ICU cognitive impairment^2,6^. Yet information about other potentially modifiable factors is inconsistent or missing^7^.

In different studies, the incidence of long-term post-ICU cognitive impairment ranged widely from 10^8^ to 57%^9^. It appeared to depend, in part, on the age of the patients^10^. This issue is particularly relevant as older patients have a higher risk of being admitted to an ICU^11^. That raises the question whether age might modulate the risk of experiencing post-ICU cognitive impairment because of its association with premorbid cognitive decline. Indeed, deterioration of cognitive function after discharge was noted in patients with pre-existing cognitive impairment^12^. However, most of the past studies lack the assessment of the patients’ pre-ICU cognitive status, hence interpretation of their cognitive outcome measures is difficult. In particular, the effect of an ICU stay on the cognitive fate of patients with a mild degree of cognitive impairment remains unclear.

In this explorative observational study, we aimed to investigate the cognitive trajectory of patients until 9 months after ICU discharge. Among other factors, we specifically addressed the issue of pre-existing cognitive impairment as a potential risk factor for post-ICU cognitive impairment. Therefore, patients were stratified by the Informant Questionnaire on Cognitive Decline in the Elderly (IQCODE)^13^. We hypothesized that a proportion of patients could suffer from long-lasting cognitive impairment, and that the factor premorbid cognitive impairment could contribute to the development of the cognitive performance in the short- and long-term after ICU treatment.

## Results

### Demographic and clinical characteristics of patient population

Out of 444 eligible patients, 108 patients (62 men and 46 women) and close relatives gave their informed consent. The following reasons prevented inclusion: informed consent could not be obtained, discharge or transfer to another hospital before study visit 1 (SV1), new diagnosis that fulfilled an exclusion criterium, and death.

**Figure 1:**
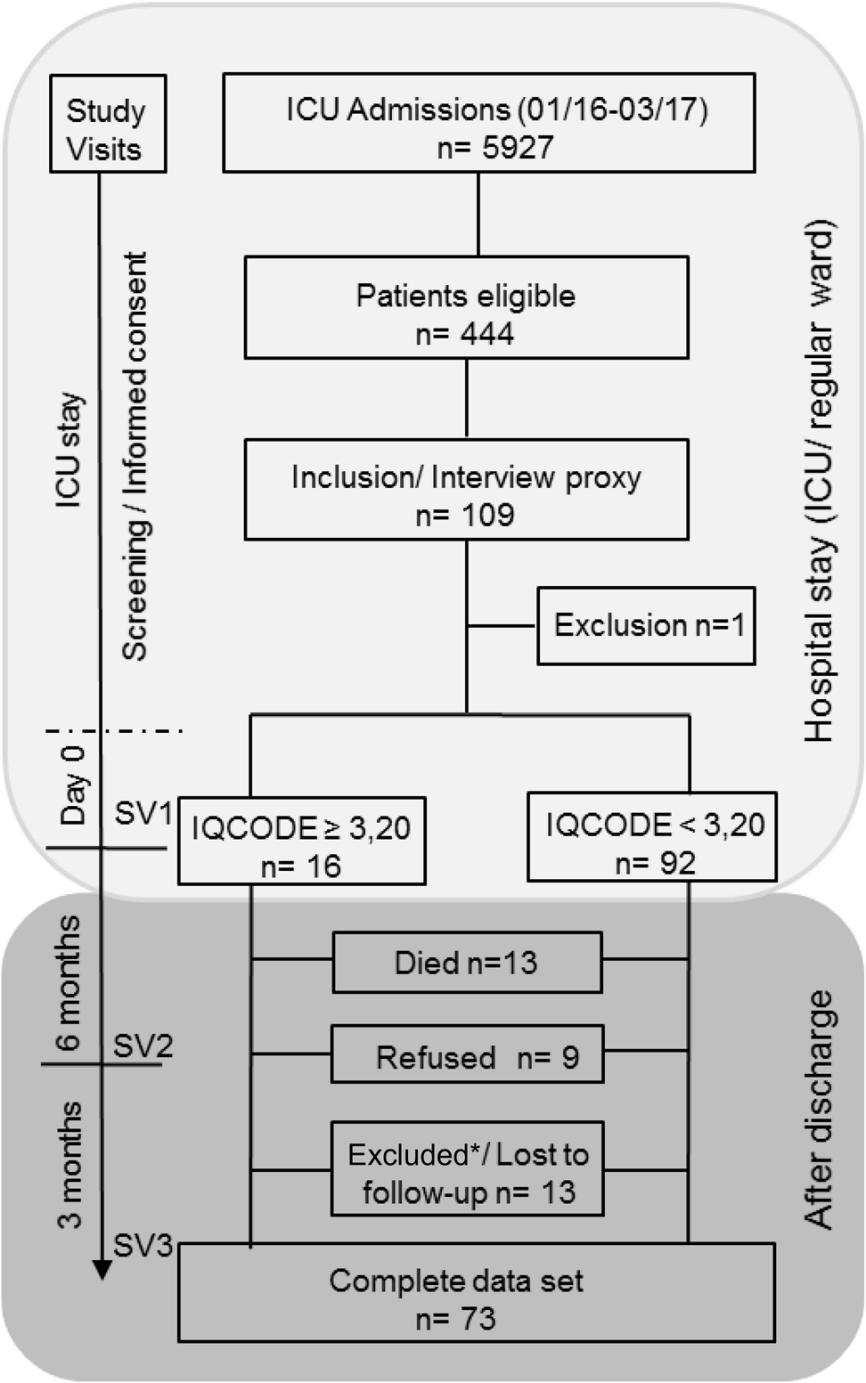
Flowchart detailing number of patients and protocol of study with timeline during and after hospital stay. Day 0 = 1 day before discharge from intensive care unit or ≤ 7 days after transfer to a regular hospital ward; ICU, intensive care unit; SV, study visit; IQCODE, Informant Questionnaire on Cognitive Decline in the Elderly; ≥ 3.20 means that there is evidence for premorbid cognitive impairment. Excluded*: exclusion because of re-admission to ICU (9 patients) and newly diagnosed stroke (1 patient).

The mean age of the study participants was 66 years ± 12 years. According to the information derived from relatives, 16 patients (15%) were likely to have developed cognitive impairment during the years prior to ICU admission (3.20 ≥ IQCODE < 3.90). The main demographic and clinical characteristics are given in Table 1. Out of 108 patients, 73 patients completed the study (68%). Reasons for discontinuation were: death, refusal, exclusion, and loss to follow-up (Fig. 1). The drop-out rate of patients with suspected premorbid cognitive impairment was 47% compared to 30% in the rest of the study group (p = 0.386, Fisher’s exact test).

**Table 1:** Characteristics of the study group and the controls.

Characteristics	Total SG	SG IQCODE < 3,20	SG IQCODE ≥ 3,20	Controls
	n = 108	n = 92	n = 16	n = 53
Age^a^	66 ± 12	64 ± 12	72 ± 10	65 ± 13
Level of education (years)^a^	12.5 ± 2	12 ± 2	12.0 ± 2	13 ± 2
Sex male	62 (57%)	56 (61%)	6 (38%)	30 (57%)
Sex female	46 (43%)	36 (39%)	10 (62%)	23 (43%)
Vascular risk score^a^	3 ± 2	3 ± 2	4 ± 1	2 ± 1
IQCODE^a^	3.10 ± 0.2	3.04 ± 0.1	3.41 ± 0.2	–
MCWT-B^a^	30 ± 5	29 ± 5	31 ± 5	31 ± 3
MCWT-B (number)	98	88	10	53
**Diagnosis ad admission-number (%)**				
After surgery	26 (24%)	20 (22%)	6 (38%)	–
Cardiac disease	25 (23%)	23 (25%)	2 (13%)	–
Respiratory disease	15 (14%)	14 (15%)	1 (6%)	–
Vascular disease	13 (12%)	8 (9%)	5 (31%)	–
Sepsis	11 (10%)	10 (11%)	1 (6%)	–
Other	18 (17%)	17 (18%)	1 (6%)	–
ICU length of stay (days)^b^	7	7	7.5	0
	3–13.5	3–13.5	3.5–14.5	–
Hospital length of stay (days)^b^	14	14	16	0
	7–21	7–20	8–24	
Charlson Comorbidity Index^a^	2 ± 2	2 ± 2	2 ± 2	–
SOFA score^a^ at admission	5 ± 4	5 ± 4	5 ± 4	–
APACHE II score^a^ at admission	18 ± 7	17 ± 7	19 ± 8	–
Duration of ventilation (hours)^b^	11.5	14.5	5	–
	1.5–101.0	2–103.0	1.5–64.5	
**Use of analgetic/sedative agent**				
Propofol^c^ (mg)^b^	170	185	85	–
	0–1766	0–2,455	0–598	
Sufentanil^c^ (µg)^b^	83	98	60	–
	0–576	0–669	0–217	
Midazolam^c^ (mg)^b^	0	2	0	–
	0–81	0–84	0–3	
**Use of analgetic or sedative agent-number (%)**				
Propofol^c^	74 (68%)	63 (69%)	11 (68%)	–
Sufentanil^c^	69 (64%)	58 (63%)	11 (68%)	–
Midazolam^c^	52 (48%)	47 (51%)	5 (31%)	–
Incidence of delirium-number (%)	23 (14%)	20 (22%)	3 (19%)	–

SG, study group; SG IQCODE ≥ 3.20, patients with suspected premorbid cognitive impairment; ^a^mean and standard deviation; ^b^median and interquartile range; ^c^medications administered at intensive care unit; number, number of patients per group who performed this test; IQCODE, Informant Questionnaire on Cognitive Decline in the Elderly; MCWT-B, Multiple Choice Word Test-B; SOFA, Sequential Organ Failure Assessment; APACHE II Score, Acute Physiology and Chronic Health Evaluation Score II; ICU, intensive care unit.

### Cognitive performance at discharge from ICU (SV1)

At the time of discharge from ICU, patients performed worse than the healthy control group in 11 of 13 tests (Supplementary Table S1).

The factor “premorbid cognitive impairment” defined as an IQCODE score between 3.20 and 3.90 showed a statistically significant relation to the CERAD Total Score (CTS) at SV1 (correlation − 0.338, p = 0.002, p-value raw, Table 2). To exclude the effect of possible premorbid cognitive impairment on the performance we also considered only patients with an IQCODE score of < 3.20. Cognitive test performance of the patients (n = 92) was significantly impaired compared to the healthy controls, with results similar to the whole study group (Supplementary Table S2).

**Table 2:** Results from linear regression models with cognitive parameters as dependent variables.

Dependent variable	Study group (N = 108)		Independent variable	Dependent variable	FU group (N = 73)		Independent variable
	Day 0				After 9 months		
	Coefficient (95% CI)	p-value			Coefficient (95% CI)	p-value	
CERAD total score	− 10 (− 17,− 4)	0.002	Premorbid cognitive decline	CERAD total score	− 10 (− 18,− 2)	0.011	Premorbid cognitive decline
	− 6 (− 12, 0)	0.040	Hospital length^a^ of stay		Dropped		Hospital length^a^ of stay
	− 5 (− 10, 0)	0.047	Delirium		− 5 (− 11, 1)	0.10	Delirium
	1 (0, 1)	< 0.001	MCWT− B		1 (0, 1)	0.013	MCWT-B
TMT A	16.5 (− 2, 35.5)	0.078	Premorbid cognitive decline	TMT A	14.5 (− 8, 36.5)	0.20	Premorbid cognitive decline
	1 (0.5, 1.5)	< 0.001	Age		1 (0.5, 1.5)	0.002	Age
	18.5 (3, 33.5)	0.018	Delirium		30 (9.5, 48)	0.004	Hospital length^a^ of stay
	7.5 (1.5, 13.5)	0.012	Midazolam^b^		Dropped		Midazolam^b^
	− 2 (− 3, − 0.5)	0.002	MCWT-B		− 1 (− 2, 0.5)	0.26	MCWT-B
TMT B	Dropped		Premorbid cognitive decline	TMT B	Dropped		Premorbid cognitive decline
	3 (2, 4)	< 0.001	Age		3 (1.5, 4)	< 0.001	Age
	7 (0, 14.5)	0.052	CCI		Dropped	0.076	Delirium
	11 (2, 20.5)	0.019	Propofol^b^		Dropped		Propofol^b^
	20 (6.5, 34)	0.019	Midazolam^b^		15.5 (5, 25.5)	0.004	Midazolam^b^
	− 4.5 (− 7, − 1.5)	0.002	MCWT-B		− 2.5 (− 6, 0.5)	0.088	MCWT-B

Dependent variables are: Consortium to Establish a Registry for Alzheimer’s Disease CERAD Total Score (lower scores indicating worse performance), Trail Making Tests (TMT) A and B in seconds (longer duration indicating worse performance); the independent variables were: premorbid cognitive impairment (according to the Informant Questionnaire on Cognitive Decline in the Elderly), Vascular Risk Score, Charlson Comorbidity Index (CCI), Sequential Organ Failure Assessment Score, intensive care unit (ICU) length of stay, hospital length of stay, incidence of delirium, duration of ventilation, duration and number of surgeries, logarithmized dosage of propofol, of sufentanil, and of midazolam, all variables were included simultaneously, covariates were age, sex, score of the Multiple Choice Word Test-B (MCWT-B) and educational level; analysis for cognitive outcome after 9 months was performed with relevant independent variables according to the analysis for Day 0 and also depression score (taken from the Hospitality Anxiety and Depression Scale).

FU group, follow-up group; C.I., confidence interval; ^a^logarithm of duration (i.e., tenfold duration of hospital stay is associated with an estimated decrease of 6 points in the CERAD Total Score); ^b^logarithm of dosage; dropped: excluded in the course of the regression model. Coefficients are unstandardized beta to allow better estimation of the effect of each independent variable on the cognitive parameters. As the coefficients are unstandardized a comparison of coefficients across different independent variables is not feasible. P-values are raw values.

In addition, we aimed to identify modifiable risk factors for cognitive impairment after ICU treatment. In a multifactorial (linear) regression model the following associations could be found: the dosage of midazolam and performance in Trail Making Test A (TMT A, p = 0.012) and Trail Making Test B (TMT B, p = 0.019), the dosage of propofol and performance in TMT B (p = 0.019), the incidence of delirium and the CTS (p = 0.047), and the hospital length of stay and the CTS (p = 0.040, all p-values are raw values, Table 2).

### Cognitive performance at 9 months after discharge from ICU (SV3)

**Figure 2:**
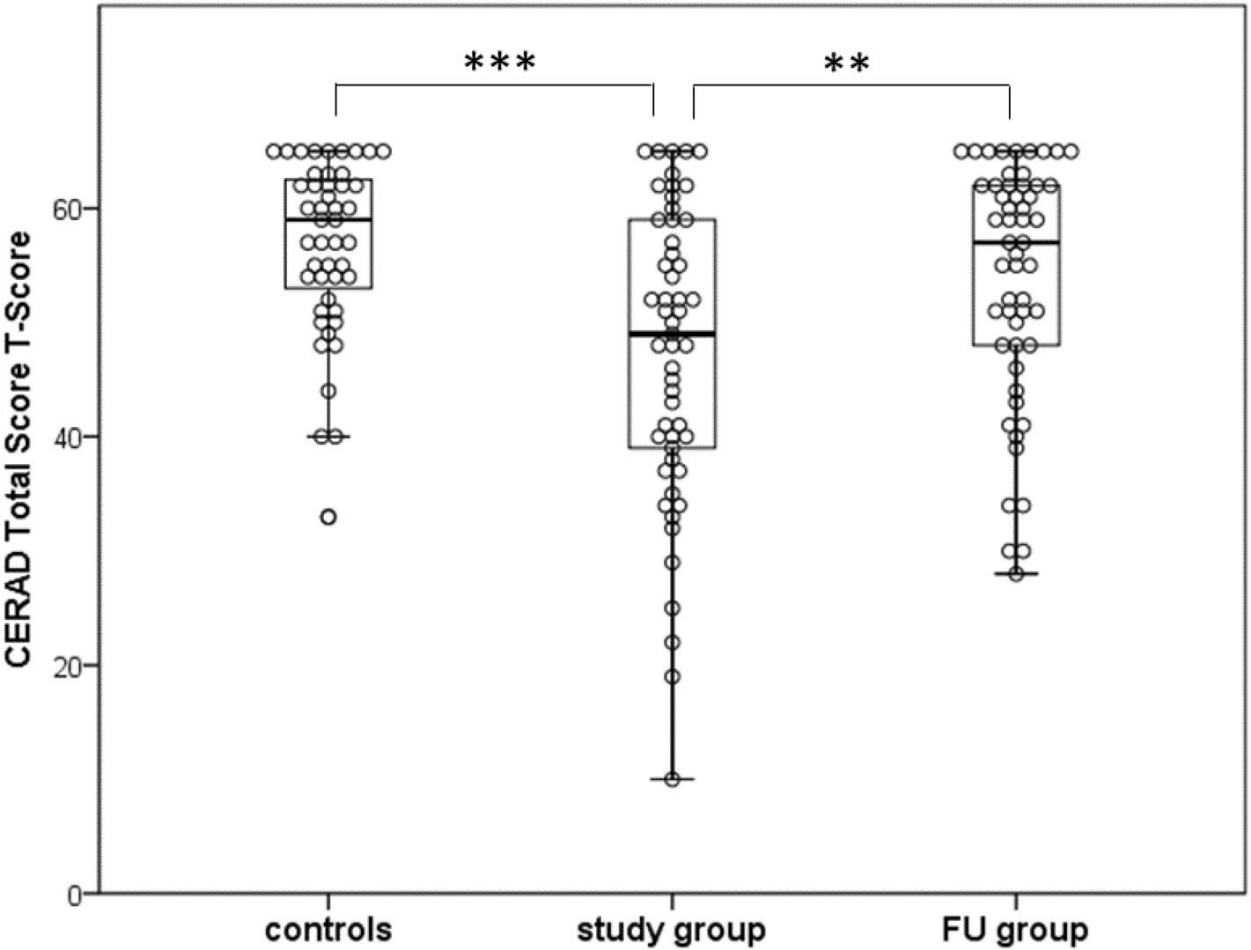
Distribution of the T-Scores of the results of the study group, the follow-up group, and the controls. Distribution of the T-Scores (transformed values of the Consortium to Establish a Registry for Alzheimer’s Disease CERAD Total Score + demographical correction factor) of the study group and FU group (subgroup without evidence of premorbid cognitive impairment, only patients who underwent both study visits), and the controls. T-scores of normative data are characterized by mean = 50 and SD = 10; maximum value is 65, minimum value is 10; normative data are available for the CERAD Total Score of subjects between 50 and 90 years of age; study group group/ FU group N = 54; controls N = 47. One point per patient/ participant of the control group; p-values: ***p < 0.001; **p < 0.01; FU group, follow-up group.

A total number of 73 patients participated at study visit 3 (SV3), 64 of them had no evidence of premorbid cognitive impairment (Supplementary Table S3). Comparison of performance of the study group at SV3 and at SV1 revealed improvement in all cognitive tests, although the statistical power in several tests was not adequate (Supplementary Table S4). The medians of the cognitive performance of the subgroup without premorbid cognitive impairment also improved at SV3 in 11 tests compared to SV1. However, comparison with the healthy controls revealed that some impairment persisted in 2 subtests of the Stroop Color and Word Test (Table 3). To evaluate long-term cognitive outcome in a second way, we additionally used the normative data available for the CTS. According to the T-Scores of the CTS, the performance of the patients without evidence of premorbid cognitive impairment improved significantly between SV1 and SV3. Three of 54 patients (6%) scored below the lower threshold of the healthy control group (Fig. 2).

**Table 3:** Results of the cognitive tests at study visits 1 and 3 of patients without evidence of premorbid cognitive impairment.

Neuropsychological tests	Study group^a^	FU group^a^	Median	p-value	Controls	Median difference	p-value
	SV1 (N = 64)	SV3 (N = 64)	Difference	Raw	(N = 53)	FU group/controls	Raw
	Median [IQR]	Median [IQR]	Median difference [95% CI]		Median [IQR]	Median difference [95% CI]	
*Verbal fluency*	19 [16, 22]	22 [17, 26]	2 [0, 4]	0.040	24 [19, 28]	− 2 [− 5, 0]	0.052
*Boston naming test*	14 [13, 15]	15 [15, 15]	0 [0, 1]	** < 0.001**	15 [14, 15]	0 [0, 1]	**0.005**
*Word list learning*	20 [16, 22]	21 [18, 24]	2 [0, 3]	0.008	23 [20, 25]	− 1 [− 2, 0]	0.18
*Word list recall*	7 [5, 9]	8 [6, 9]	1 [0, 2]	0.048	8 [7, 9]	0 [− 1, 1]	0.68
*Word list recognition discriminability*	20 [19, 20]	20 [20, 20]	0 [0, 0]	0.086	20 [20, 20]	0 [0, 0]	0.63
*Constructional praxis*	10 [8, 10]	10 [9, 11]	0 [0, 1]	0.21	10 [10, 11]	0 [− 1, 0]	**0.034**
*Praxis recall*	8 [6, 10]	9 [8, 10]	1 [0, 1]	0.063	9 [8, 10]	0 [− 1, 0]	0.57
*MMSE*	28 [26, 29]	29 [28, 30]	1 [1, 2]	** < 0.001**	29 [28, 30]	0 [0, 0]	0.88
*Trail making test A*	46 [34, 69]	40 [33, 57.5]	− 6 [0, − 13]	0.065	35 [28, 49]	5 [0, 10]	0.051
*Trail making test B*	116 [86, 174]	90 [73, 126]	− 23 [− 7, − 40]	**0.003**	78 [61, 112]	11 [− 3, 23]	0.12
***Colorwords reading***	39.5 [35, 47.5]	33.5 [30, 41.5]	− 5 [− 2, − 8]	**0.001**	31 [27, 35]	3 [1, 6]	**0.010**
***Naming color patches***	54 [46, 73]	50 [43, 63]	− 6 [− 1, − 11]	0.024	48 [41, 53]	3 [− 1, 8]	0.098
***Color-word condition***	115 [93, 159]	100.5 [80.5, 121.5]	− 21.5 [− 8, − 35]	**0.002**	91 [80, 101]	10 [1, 20]	**0.030**

Comparison of the results of study visit 1 and 3 and comparison of follow-up group to controls; ^a^patients who underwent study visits 1 and 3; FU group, follow-up group; MMSE, Mini Mental Status Examination; IQR, interquartile range; C.I., confidence interval; SV1 (3), study visit 1 (3); Italics: subtests of the Consortium to Establish a Registry for Alzheimer’s Disease CERAD Plus battery; Bold italics: subtests of the Stroop Color and Word Test. The p-values marked in bold are still significant after correction (Bonferroni-Holm).

Nine patients of the follow-up group (FU group) were suspected to have cognitive impairment prior to ICU admission according to the IQCODE. The cognitive performance of these 9 patients improved numerically after 9 months in the majority of the tests (Supplementary Table S5). However, there was no improvement in the TMT B. To explore this observation further, we compared the longitudinal cognitive changes between SV1 and SV3 of two matched patient groups with and without evidence of premorbid cognitive impairment. Only 2 out of 9 patients with evidence of premorbid cognitive impairment, but 7 out of 9 patients without evidence of premorbid cognitive impairment improved their performance in TMT B (Supplementary Fig. S1). We also used the T-Scores of the CTS to evaluate the development of the cognitive performance over 9 months for both matched subgroups with and without premorbid cognitive impairment in a descriptive way. Regarding the means of the T-Scores the patients with evidence of premorbid cognitive impairment started from a lower level (mean 36, SD 15) and stayed at a lower level in study visit 3 (mean 46, SD 13) than the other patients (study visit 1: mean 46, SD 14, study visit 3: mean 52, SD 11). The distribution of the CTS T-Scores of both subgroups at study visit 1 and 3 is shown in Supplementary Fig. S2.

Furthermore the factor “premorbid cognitive impairment” showed again a strong association with the CTS at SV3 (p = 0.011, p-value raw, Table 2) in a linear regression model.

#### Psychiatric morbidity and early risk factors for cognitive impairment at 9 months after discharge from ICU

At SV3, according to the Hospitality Anxiety and Depression Scale (HADS), 4 patients out of 73 fulfilled the criteria for depression. Correlations could be found between the HADS Score Depression and the cognitive parameters CTS, TMT A and TMT B (Supplementary Fig. S3).

We examined the effect of the independent factors which had demonstrated an association with cognitive outcome at day 0. In addition, the HADS Depression Score was considered. This analysis revealed that midazolam was related to the performance in TMT B at SV3 (p = 0.004). Furthermore, the initial hospital length of stay was associated with the performance in TMT A (p = 0.004, p-values are raw values, Table 2).

## Discussion

In this prospective observational study, we detected acute cognitive impairment in patients after ICU treatment. After 9 months the cognitive performance improved significantly, but 6% of the patients remained cognitively impaired. Special consideration was given to patients with evidence of premorbid cognitive impairment. By integrating information about the premorbid cognitive state, the present results add knowledge to the few prospective studies addressing the impact of ICU treatment on people who are at high risk for the development of a dementing disorder. To our knowledge Cog-I-CU is the first study that aimed to detect a premorbid intermediate level of cognitive decline in ICU patients in a retrospective way.

Our results demonstrate that cognitive function was impaired at the time of discharge from ICU. The mean age of patients (66 years) indicated that findings may have been heavily influenced by patients of older age. This observation is important as the prevalence of cognitive impairment increases with age. In a recent review Rengel and colleagues^14^ reported that baseline cognitive impairment is a risk factor for cognitive impairment following critical illness. To account for the possibility that cognitive impairment at SV1 may simply reflect a higher prevalence of a premorbid state, patients were stratified by the IQCODE^13^. Cognitive function was found to be impaired even in patients without evidence of premorbid cognitive impairment after discharge.

After 9 months the cognitive performance of these patients had improved although slight numerical differences compared to the controls persisted in some tests. These findings appear to indicate a better prognosis than in previous investigations in long-term survivors of critical illness^1–3,8,9^. The discrepancy probably resulted from (i) a comparatively milder disease severity of the included patients, (ii) a lower incidence of delirium (25% in the present study cohort compared to 75%^2^, 78% and 67%^15^, 46%^16^, and 56%^17^, in previous reports) and from the fact that the proportion of patients who finished the study was biased into a healthier state because several participants were lost between initial discharge and SV3 due to death (N = 13) or re-admission to ICU (N = 9).

According to the CTS and compared to the healthy control group long-term cognitive impairment was detected in three patients (6%). Because of the small number no firm conclusions can be drawn, but different possible explanations are to discuss. With this caveat, we believe our results to suggest that cognitive impairment after ICU treatment is long-lasting or even persistent. In 2005 nearly two million people were admitted to an ICU in Germany^18^. The rate of ICU treatment during hospitalization increases every year^19^. Therefore, if the rate of cognitive impairment were to be confirmed in a larger cohort this would mean a considerable additional burden of long-term cognitive impairment for this population. Alternatively, ongoing chronic medical illness, or interim events, such as re-admission to a hospital between SV1 and SV3 may have played a role. A relevant contribution of hospitalization to the rate of cognitive decline is supported by observations in a cohort of nearly 800 older people without dementia^12^.

We confirmed previous evidence^2,20,21^ that delirium is a predictor for worse cognitive outcome after discharge from ICU. Furthermore, midazolam correlated with executive dysfunction as evidenced by the results in the Trail Making Tests. At first sight, this finding may be surprising as sedatives did not appear to influence cognitive outcome in previous studies^22,23^. However, because administration of benzodiazepines is associated with the presence of delirium^24–26^ and benzodiazepines are also used to treat delirium, the effect of midazolam may be indirect and be fully explained by this association. This suggestion is supported by a multicentre study with 1,048 ICU patients. In the majority of delirium days delirium was associated with sedatives^27^.

Because patients were treated for a large variety of different medical or surgical conditions, it seems unlikely that cognitive impairment originated from a single pathogenic factor. Instead, noxious factors common to several diverse medical conditions are implicated. One such factor may be the biological stress response^10^. The duration of treatment is likely related to the duration and magnitude of the biological stress response. Thus, an association between the duration of stay and the cognitive outcome can be expected. However, there was no relevant correlation between the cognitive outcome and the ICU length of stay. This is in accordance to previous studies^8,28,29^. Hopkins and colleagues reported worse cognitive outcome if the duration of the ICU stay exceeded 27 days^30^. Due to the relatively short median length of the ICU stay in the present study the factor explained above may not have been fully effective. Interestingly, the hospital length of stay was related to the cognitive outcome, so the concept of a pathogenic stressful environment should not be discarded for inpatient treatment in general.

Cognitive functions also improved after 9 months in patients with an intermediate IQCODE score, a score that may correspond to mild cognitive impairment (MCI). Although this cohort of patients was small, it is of special interest, because any further cognitive decline in these patients would likely have the most significant functional consequences. While most of the matched subgroup of patients without evidence of premorbid cognitive impairment improved numerically in TMT B, the majority of patients with evidence of premorbid cognitive impairment did not recover. One possible explanation for this observation may be that a dementing disorder that started before the ICU admission has been accelerated. As in patients with MCI who later convert to Alzheimer’s disease, decline of executive functions follows the onset of memory dysfunction^31^, in this cohort executive function may be especially vulnerable toward ICU treatment associated injury. Addressing these issues will require larger patient cohorts and longer observation periods.

In our study, 4 out of 73 patients fulfilled the criteria for a depressive disorder according to the HADS 9 months after discharge from ICU, which exceeds the annual incidence of 1–2/100 of the general German population^32^. The HADS score for depression was related to a worse cognitive outcome at SV3. It is difficult to distinguish whether depression is accompanied or caused by cognitive impairment. One prospective cohort showed that executive dysfunction at 3 months after ICU discharge remained associated with the severity of depressive symptoms even after adjustment for depression at baseline^33^. Future studies should focus on possible common neurobiological patterns for effects on mood or cognition.

We consider it a strength of the study that the assessment of the premorbid cognitive status by a validated interview with a proxy allowed to perform pre-planned analyses with patients in whom cognition was likely premorbidly impaired. As the interviews were conducted with a proxy in person an overestimation of the premorbid cognitive abilities of the patients cannot be excluded.

Also we minimised bias by adjusting for known or supposed confounders, although the effects of different variables cannot be fully separated.

This study was monocentric, so that one should be careful when extrapolating our results to other patient populations. The small sample size is a major limitation and may probably mean that statistical differences were not recognized due to a lack of statistical power. Also clinically relevant effects may not be reproduced in a larger sample. Especially the results of the subgroup of patients with suspected premorbid cognitive impairment have to be interpreted with caution as only nine datasets were available for final analysis. On the other hand the percentage of patients with suspected premorbid cognitive impairment appears representative. Our observations may inspire future studies to focus on this vulnerable cohort but they have to be reproduced in a study with a larger sample size to draw firm conclusions. A clear advantage of the monocentric design is the reduced variability in neuropsychological assessment as only a uniformly trained small group of study staff members was involved.

The incidence of delirium could only be estimated indirectly from case notes. It is also difficult to discriminate the effects of the ICU stay from the hospitalization itself. This may partially be addressed by including a matching non-ICU inpatient cohort for comparison in future trials.

## Conclusion

Cog-I-CU has provided new evidence on long-term cognitive impairment after ICU treatment impacting both patients with and without premorbid cognitive impairment. Our results suggested that cognitive functions may be particularly vulnerable in patients with suspected premorbid cognitive impairment. Due to the small sample size the observations have to be confirmed in a larger cohort. Future studies should investigate whether ICU treatment may accelerate cognitive decline from intermediate levels of cognitive impairment towards dementia. Enhanced risk for future cognitive decline may need to be included in treatment decisions. Future studies should also aim to investigate factors that may enhance resilience toward ICU-related cognitive impairment.

## Patients and methods

### Study design

“Long-term cognitive impairment after ICU treatment” (Cog-I-CU) was an explorative, single-center, longitudinal and observational cohort study (DRKS00011162). All patients admitted to the medical and surgical ICU of the University of Leipzig Medical Center between January 2016 and March 2017 were screened. At the time of enrollment all patients or their legal representatives gave written informed consent. Clinical characteristics were collected prospectively until discharge. After the ICU management, three study visits were performed: SV1 (day 0) at 1 day before discharge from ICU or within a maximum of 7 days after transfer to a regular hospital ward; study visit 2 (SV2) at 6 months after day 0; and SV3 at 9 months after day 0 (between 250 and 305 days after SV1). Cognitive function tests were performed at SV1 and SV3. At SV2 a structured telephone interview was conducted in which important events with potential influence on cognitive outcome after 9 months were obtained.

The study was approved by the local Ethics Committee of the University of Leipzig (09/15; 314-15-24082015). In addition, all methods were performed in accordance with the relevant guidelines and regulation.

### Study population

Adult patients admitted to an ICU for non-neurological conditions were considered for inclusion. Exclusion criteria were: history of stroke within the last 6 months or stroke at any time in the past with known permanent cognitive impairment, traumatic brain injury, neurological or neurodegenerative conditions or a known medical diagnosis that might confound cognitive function test outcome including liver cirrhosis, end-stage renal failure, current chemotherapy for malignant disease, psychiatric disorders; treatment in an ICU for ≥ 7 days during the last 6 months before enrollment; care in nursing homes at the time of enrollment, estimated probability of survival in 1 year < 50%, language barrier or physical conditions that prevent a reliable assessment.

In particular, patients with suspected but undiagnosed dementia according to the survey with a close relative (IQCODE ≥ 3.90, see below) were excluded. Between SV1 and SV3 the following conditions led to termination of the study for a patient: new ICU stay after initial discharge from hospital, newly diagnosed non-neurodegenerative brain disease such as stroke, medical conditions (e.g., traumatic injuries) hindering the conduction of SV3, and death.

### Healthy control group

To ensure a reliable analysis of the cognitive performance of patients of various ages, we established a control group of healthy subjects according to the exclusion criteria of the study group. A total of 53 subjects were recruited (30 men and 23 women; mean age 65 ± 13 years, Table 1).

### Premorbid cognitive status

Information about the premorbid cognitive function was derived from the IQCODE, a validated screening tool about the daily cognitive performance^13^. Based on a 5-point scale from “much improved” to “much worse” the threshold for suspicious cognitive impairment ranges from 3.20 to 3.90 in the literature^2,34^. In a study on German patients those with MCI were reliably distinguished from healthy subjects by the German version of the IQCODE at a cutoff-score of 3.20 (sensitivity 60%, specifity 78%)^34^. Based on this investigation we considered patients with an IQCODE < 3.20 for the comparison of the cognitive performance with the control group to minimize the risk of a significant premorbid decline. IQCODE was presented to relatives familiar with the patient. An IQCODE score of ≥ 3.90 indicates a high probability of dementia^2^.

### Neuropsychological assessment

This was assessed by the validated test battery of the Consortium to Establish a Registry for Alzheimer’s Disease (CERAD)^35^. By its design, it offers the possibility to measure *global cognition* in the clinical context. Normative data are available for the CERAD Total Score (CTS) of subjects between 50 and 90 years of age. To compare the CTS with the normative data, the scores were transformed into T-Scores^36^.

The Plus-version of the CERAD test battery also includes the Trail Making Test parts A and B (TMT A and B) covering the domains *attention, executive function and cortical processing speed*. The Stroop Color and Word Test including three subtests was used for further evaluation of *executive function*^37^*.*

To estimate the level of *premorbid intelligence* we used the Multiple Choice Word Test-B (MCWT-B), a validated one-scale verbal based intelligence test^38^ with known applicability in ICU survivors^29^. The MCWT-B is based on the assumption that verbal knowledge is not or only minimally affected by acute brain injury^39^.

### Psychiatric assessment

This was assessed at 9 months after discharge from ICU using the Hospitality Anxiety and Depression Scale (HADS)^40^. The scores for depression were also considered in correlation analyses.

### Statistical analysis

The study cohort was described by mean (standard deviation) for continuous characteristics, number (percent) for categorical and by median [interquartile range] for characteristics with skewed distribution. Accordingly, groups were compared by Wilcoxon-Mann–Whitney U-test. Nonparametric 95% confidence intervals were calculated following the method of Hodges and Lehmann.

Linear mixed models were applied for two purposes: First, we looked for potentially confounding covariates at SV1. We started with a priori chosen variables based on clinical judgement and previous research^2^. These were: Premorbid cognitive impairment (determined by the IQCODE), Vascular Risk Score, Sequential Organ Failure Assessment Score (SOFA), Charlson Comorbity Index (CCI), ICU length of stay, hospital length of stay (duration between admission and the day of SV1), duration of mechanical ventilation, duration of surgeries, number of surgeries, logarithmized dosages of propofol, midazolam and sufentanil, and incidence of delirium (Supplementary Text). The incidence of delirium was estimated indirectly from the case notes of the treating physicians. The cognitive outcome variables CTS, TMT A and B were chosen as dependent variables. Afterwards, we reduced the model by backward exclusion on the basis of the Akaike information criterion. Second, we applied linear mixed models to adjust for known confounders, age, sex, and MCWT-B. The models were reduced, if possible, to get scarce models. We estimated effects including confidence intervals.

According to a German study^34^, an IQCODE score of 3.20 may differentiate between healthy subjects and those with MCI. Therefore, the study group was stratified into patients with an IQCODE < 3.20, and patients with an IQCODE ranging from 3.20 to 3.89 who were suspected to have cognitive impairment but unlikely to have frank dementia.

We checked the depression score of the Hospital Anxiety and Depression Scale (HADS-D) visually for correlations with selected cognitive parameters and calculated Spearman's rank correlation coefficient.

The general significance level was set at α = 5% for two-tailed testing. Bonferroni-Holm correction for multiple comparisons was used as appropriate. Data preparation, descriptive statistics, correlation analysis and nonparametric tests were performed with IBM SPSS Statistics, version 24. All other analyses were done with R including the packages *Hmisc*, *lmerTest*, *ggplot2* and *grDevices*^41^*.*

## Supplementary information

Supplementary information.

## Data Availability

The datasets generated during and/or analysed during the current study are available from the corresponding author on reasonable request.
