# Supplementary material for: Long-term cognitive impairment after ICU treatment: a prospective longitudinal cohort study (Cog-I-CU)
Source: Sci Rep. 2020 Sep 23;10:15518. doi: 10.1038/s41598-020-72109-0 (PMC7511316; doi:10.1038/s41598-020-72109-0)
Supplement: Supplementary file 1 — Supplementary information. [file 41598_2020_72109_MOESM1_ESM.pdf]

Supplement to: Long-term Cognitive Impairment after ICU Treatment–

a prospective longitudinal cohort study (Cog-I-CU)

Annekatrin Müller[1]*, MD; Judith von Hofen-Hohloch[1], MD; Meinhard Mende[2], DSc; Dorothee Saur[1], MD; Christopher Fricke[1], MD; Sven Bercker[3], MD; Sirak Petros, MD[4];

and Joseph Classen[1], MD

[1]Department of Neurology, University of Leipzig Medical Center, Leipzig, Germany

[2]Institute for Medical Informatics, Statistics and Epidemiology, University of Leipzig, Leipzig, Germany

[3]Department of Anaesthesiology and Intensive Care Medicine, University of Leipzig Medical Center, Leipzig, Germany.

[4]Medical Intensive Care Unit, University of Leipzig Medical Center, Leipzig, Germany

Corresponding author: Annekatrin Müller

University of Leipzig Medical Center, Department of Neurology, Liebigstr. 20, 04103 Leipzig, Germany; Tel.: +49 341 97 24200, FAX +49 341 97 24209;

e-mail: annekatrin.mueller@medizin.uni-leipzig.de

Supplementary Table S1 Results of study visit 1, whole study group, comparison of study group to controls

	study group SV1 N=108	controls N= 53	Median Difference	p- value raw	p- value adjusted
	Median [IQR]	Median [IQR]	Med. Diff. [95% C.I.]		
Verbal Fluency	18 [14, 22]	24 [19, 28]	-5 [-7, -3]	< 0.001	**< 0.001**
Boston Naming Test	14 [13, 15]	15 [14, 15]	0 [0, 0]	0.16	0.16
Word List Learning	19 [15, 22]	23 [20, 25]	-3 [-5, -2]	< 0.001	**< 0.001**
Word List Recall	6 [4, 8]	8 [7, 9]	-1 [-2, 0]	0.001	**0.003**
Word List Recognition Discriminability	20 [19, 20]	20 [20, 20]	0 [0, 0]	0.063	0.13
Constructional Praxis	9 [8, 10]	10 [10, 11]	-1 [-1, 0]	< 0.001	**< 0.001**
Praxis Recall	8 [5, 9]	9 [8, 10]	-1 [-2, 0]	< 0.001	**< 0.001**
MMSE	27,5 [25, 29]	29 [28, 30]	-1 [-2,-1]	< 0.001	**< 0.001**
Trail Making Test A (TMTA)	53 [39, 82]	35 [28, 49]	18 [11, 25]	< 0.001	**< 0.001**
Trail Making Test B (TMTB)	135 [95, 287]	78 [61, 112]	50 [32, 73]	< 0.001	**< 0.001**
Colorwords Reading	40 [35, 49]	31 [27, 35]	9 [7, 12]	< 0.001	**< 0.001**
Naming Color Patches	57 [49, 78]	48 [41, 53]	11 [7, 17]	< 0.001	**< 0.001**
Color-Word Condition	134 [101, 174]	91 [80, 101]	38 [25, 54]	< 0.001	**< 0.001**

MMSE, Mini Mental Status Examination; IQR, interquartile range; C.I., confidence interval; SV1, study visit 1; Light grey: subtests of the Consortium to Establish a Registry for Alzheimer´s Disease CERAD Plus battery; Dark grey: subtests of the Stroop Color and Word Test

Supplementary Table S2 Results of study visit 1, patients without evidence of premorbid cognitive impairment and comparison of study group to controls

	study group SV1[a] N= 92	controls N= 53	Median Difference	p- value raw	p- value adjusted
	Median [IQR]	Median [IQR]	Med. Diff. [95% C.I.]		
Verbal Fluency	18 [15, 22]	24 [19, 28]	-5 [-7, -3]	< 0.001	**< 0.001**
Boston Naming Test	14 [13, 15]	15 [14, 15]	0 [0, 0]	0.44	0.44
Word List Learning	20 [16, 22]	23 [20, 25]	-3 [-5, -2]	< 0.001	**< 0.001**
Word List Recall	7 [5, 9]	8 [7, 9]	-1 [-2, 0]	0.015	**0.044**
Word List Recognition Discriminability	20 [19, 20]	20 [20, 20]	0 [0, 0]	0.29	0.58
Constructional Praxis	9 [8, 10]	10 [10, 11]	-1 [-1, 0]	< 0.001	**< 0.001**
Praxis Recall	8 [6, 10]	9 [8, 10]	-1 [-2, 0]	0.002	**0.008**
MMSE	28 [26, 29]	29 [28, 30]	-1 [-2,-1]	< 0.001	**0.001**
Trail Making Test A (TMTA)	49.5 [38, 71]	35 [28, 49]	14 [8, 21]	< 0.001	**< 0.001**
Trail Making Test B (TMTB)	126 [89, 206]	78 [61, 112]	42 [25, 62]	< 0.001	**< 0.001**
Colorwords Reading	39.5 [35, 46]	31 [27, 35]	9 [6, 11]	< 0.001	**< 0.001**
Naming Color Patches	57 [48, 71]	48 [41, 53]	10 [6, 14]	< 0.001	**< 0.001**
Color-Word Condition	126 [93, 170]	91 [80, 101]	35 [22, 50]	< 0.001	**< 0.001**

SV1, study visit 1, [a] patients without evidence of premorbid cognitive impairment; MMSE, Mini Mental Status Examination; IQR, interquartile range; C.I., confidence interval; Light grey: subtests of the Consortium to Establish a Registry for Alzheimer´s Disease CERAD Plus battery; Dark grey: subtests of the Stroop Color and Word Test. The p-values marked in bold are still significant after correction (Bonferroni-Holm).

Supplementary Table S3 Demographic and clinical characteristics of the follow-up group and subgroups of the follow-up group

Characteristics	FU group<br>n=73	FU group IQCODE < 3.20<br>n=64	FU group IQCODE ≥ 3.20<br>n=9	FU subgroup IQCODE < 3.20<br>n=9
Age[a]	63 ± 12.5	64 ± 12	74 ± 12	73 ± 9
Level of education (years)[a]	12.5 ± 2	12.5 ± 2	12.5 ± 2.5	12.5 ± 2
Sex male	45 (62%)	48 (67%)	3 (33%)	4 (44%)
Sex female	28 (38%)	24 (33%)	6 (67%)	5 (56%)
Vascular Risk score[a]	3 ± 2	3 ± 2	4 ± 2	3 ± 1
IQCODE[a]	3.09 ± 0.2	3.04 ± 0.1	3.40 ± 0.2	3.07 ± 0.1
MCWT-B[a]	30 ± 5	29 ± 5	31 ± 5	31 ± 3
MCWT-B (number)	70	70	8	9
Diagnosis ad admission- number (%)				
After surgery	20 (27%)	17 (24%)	4 (44%)	2 (22%)
Cardiac disease	16 (22%)	18 (25%)	1 (11%)	4 (44%)
Respiratory disease	9 (12%)	10 (14%)	1 (11%)	0
Vascular disease	8 (11%)	5 (7%)	3 (33%)	1 (11%)
Sepsis	7 (10%)	7 (10%)	0	1 (11%)
Other	13 (17 %)	15 (20%)	0	1 (11%)
ICU length of stay (days)[b]	7.5	8	7.5	4.5
	2.5 -- 14.5	3 -- 14.5	3 -- 14.5	2 -- 17
Hospital length of stay (days)[b]	13	14	14	9
	7 -- 22	7 -- 22	8 -- 21	6 -- 20.5
Charlson Comorbidity Index[a]	2 ± 2	2 ± 2	2 ± 2	2 ± 2
SOFA Score[a] at admission	4 ± 4	5 ± 4	4 ± 5	4 ± 4
APACHE II score* at admission	16 ± 7	17 ± 7	16 ± 9	17 ± 9
Duration of ventilation (hours)[b]	11.0	17.5	5.5	34.5
	0.5 -- 101.0	1.5 -- 112.0	0 -- 64.5	0 -- 108
Use of analgetic/sedative agent				
Propofol[c] (mg)[b]	160	175	110	90
	0 -- 2517	0 -- 3269	0 -- 598	0 -- 2924
Sufentanil[c] (µg)[b]	80	98	60	130
	0 -- 956	0 -- 1057	0 -- 202	0 -- 2034
Midazolam[c] (mg)[b]	2	3	0	30
	0 -- 81	0 -- 94	0 -- 3	0 -- 135
Use of analgetic or sedative agent - number (%)				
Propofol[c]	49 (67%)	49 (68%)	5 (56%)	6 (56%)
Sufentanil[c]	45 (62%)	45 (62%)	3 (33%)	5 (56%)
Midazolam[c]	38 (53%)	39 (54%)	6 (67%)	5 (56%)
Incidence of delirium - number (%)	18 (25%)	18 (25%)	2 (25%)	2 (22%)

≥ 3.20 means evidence of premorbid cognitive impairment; FU subgroup IQCODE < 3.20 means patients of the follow-up group without evidence of premorbid cognitive impairment matched to the subgroup of patients with evidence of premorbid cognitive impairment in age, sex and educational level; [a] mean and standard deviation; [b] median and interquartile range; [c] medications administered at ICU; number, number of patients per group who performed this test; IQCODE, Informed Questionnaire on Cognitive Decline in the Elderly; SOFA, Sequential Organ Failure Assessment; APACHE II Score, Acute Physiology and Chronic Health Evaluation Score II; ICU, intensive care unit

Supplementary Table S4 Descriptive data of the results of patients (whole study group) in study visit 1 compared to their results in study visit 3, comparison follow-up group to controls

	study group[a] SV1 (N=73)	FU group[a] SV3 (N=73)	Median Difference	p- value raw	p- value adjusted	controls N= 53	Median Difference FU group/ controls	p- value raw
	Median [IQR]	Median [IQR]	Med. Diff. [95% C.I.]			Median [IQR]	Med. Diff. [95% C.I.]	
Verbal Fluency	19 [15, 22]	21 [17, 26]	3 [0, 4]	0.017	0.12	24 [19, 28]	-3 [-5, 0]	0.026
Boston Naming Test	14 [14, 15]	15 [14, 15]	0 [0, 1]	< 0.001	**0.005**	15 [14, 15]	0 [0, 0]	0.034
Word List Learning	19 [15, 22]	21 [18, 24]	2 [1, 3]	0.007	0.063	23 [20, 25]	-2 [-3, 0]	0.041
Word List Recall	6 [4, 9]	8 [6, 9]	1 [0, 2]	0.050	0.15	8 [7, 9]	0 ['-1, 0]	0.18
Word List Recognition Discriminability	20 [19, 20]	20 [20, 20]	0 [0, 0]	0.17	0.17	20 [20, 20]	0 [0, 0]	0.72
Constructional Praxis	10 [8, 10]	10 [9, 11]	0 [0, 1]	0.048	0.19	10 [10, 11]	0 [-1, 0]	0.057
Praxis Recall	8 [6, 10]	9 [7, 10]	1 [0, 2]	0.034	0.17	9 [8, 10]	0 [-1, 0]	0.27
MMSE	28 [25, 29]	29 [28, 30]	1 [1, 2]	< 0.001	**< 0.001**	29 [28, 30]	0 [-1, 0]	0.49
Trail Making Test A (TMTA)	50 [36, 71.5]	41 [33, 62.5]	-6 [-13, 0]	0.055	0.13	35 [28, 49]	7 [2, 13]	0.013
Trail Making Test B (TMTB)	120 [88.5, 199.5]	94 [75, 132.5]	-22 [-39, -5]	0.007	0.056	78 [61, 112]	16 [2, 29]	0.023
Colorwords Reading	39 [35, 49]	34 [30, 42]	-5 [-8, -2]	0.002	**0.022**	31 [27, 35]	4 [1, 6]	**0.002**
Naming Color Patches	56 [47, 78]	52 [43, 64]	-6 [-11, -1]	0.024	0.14	48 [41, 53]	4 [0, 9]	0.032
Color-Word Condition	117 [98, 167.5]	103 [82, 123.5]	-21 [-34, -7]	0.002	**0.020**	91 [80, 101]	12 [3, 22]	0.008

[a] patients who underwent study visits 1 and 3; FU group, follow-up group; MMSE, Mini Mental Status Examination; IQR, interquartile range; C.I., confidence interval; SV1 (3), study visit 1 (3); Light grey: subtests of the Consortium to Establish a Registry for Alzheimer´s Disease CERAD Plus battery; Dark grey: subtests of the Stroop Color and Word Test. The p-values marked in bold are still significant after correction (Bonferroni-Holm).

Supplementary Table S5 Results of the cognitive tests at study visits 1 and 3 of patients with evidence of premorbid cognitive impairment (cog imp)

Neuropsychological Tests	SG group [a] cog imp SV1 (N=9)	FU group [a] cog imp SV3 (N=9)	Median Difference	p- value raw	controls (N= 9)	Median Difference FU group/ controls	p- value raw
	Median [IQR]	Median [IQR]	Median Difference [95% C.I.]		Median [IQR]	Median Difference [95% C.I.]	
Verbal Fluency	14 [11, 20]	18 [14, 25]	4 [-1, 10]	0.14	21 [19, 27]	-4 [-10, 4]	0.26
Boston Naming Test	14 [14, 14]	14 [13, 15]	0 [-1, 1]	0.80	14 [14, 15]	0 [-1, 1]	0.67
Word List Learning	15 [13, 18]	16 [14, 21]	1 [-3, 5]	0.61	21 [19, 23]	-4 [-8, 0]	0.063
Word List Recall	3 [2, 6]	4 [3, 7]	1 [-1, 3]	0.26	7 [5, 9]	-2 [-5, -1]	**0.019**
Word List Recognition Discriminability	19 [18, 20]	18 [18, 20]	0 [-2, 1]	0.61	19 [19, 20]	-1 [-2, 0]	0.19
Constructional Praxis	8 [8, 10]	10 [10, 11]	2 [0, 3]	**0.040**	10 [10, 11]	0 [-1, 0]	0.93
Praxis Recall	6 [4, 6]	7 [3, 10]	1 [-3, 4]	0.44	10 [8, 11]	-3 [-6, 0]	0.050
MMSE	25 [21, 27]	28 [26, 29]	2 [0, 6]	0.077	28 [27, 30]	-1 [-3, 1]	0.34
Trail Making Test A	77 [52, 137.5]	70 [43.5, 111.5]	-9 [-60, 33]	0.61	35 [28, 56]	23 [-2, 74]	0.11
Trail Making Test B	168 [106, 296]	169 [116, 273]	-1 [-83, 75]	0.86	97 [57.5, 121.5]	72 [18, 186]	**0.014**
Colorwords Reading	39 [35.5, 62.5]	42 [33, 65]	-4 [-19, 15]	0.67	36 [32, 40.5]	6 [-3, 30]	0.19
Naming Color Patches	77 [49, 101]	62 [49, 91]	-6 [-38, 17]	0.73	50 [45.5, 59]	12 [-2, 39]	0.094
Color-Word Condition	146 [100.5, 184.5]	105 [100.5, 224.5]	-5 [-70, 78]	0.93	101 [85.5, 117]	14 [-15, 108]	0.30

Comparison of results in study visit 1 and 3 and comparison of follow-up group to an age, sex- and education- matched subgroup of controls; SG, study group; FU group, follow-up group, [a] patients who underwent study visits 1 and 3; MMSE, Mini Mental Status Examination; IQR, interquartile range; C.I., confidence interval; SV1 (3), study visit 1 (3) Light grey: subtests of the Consortium to Establish a Registry for Alzheimer´s Disease CERAD Plus battery; Dark grey: subtests of the Stroop Color and Word Test. All p-values are raw values.

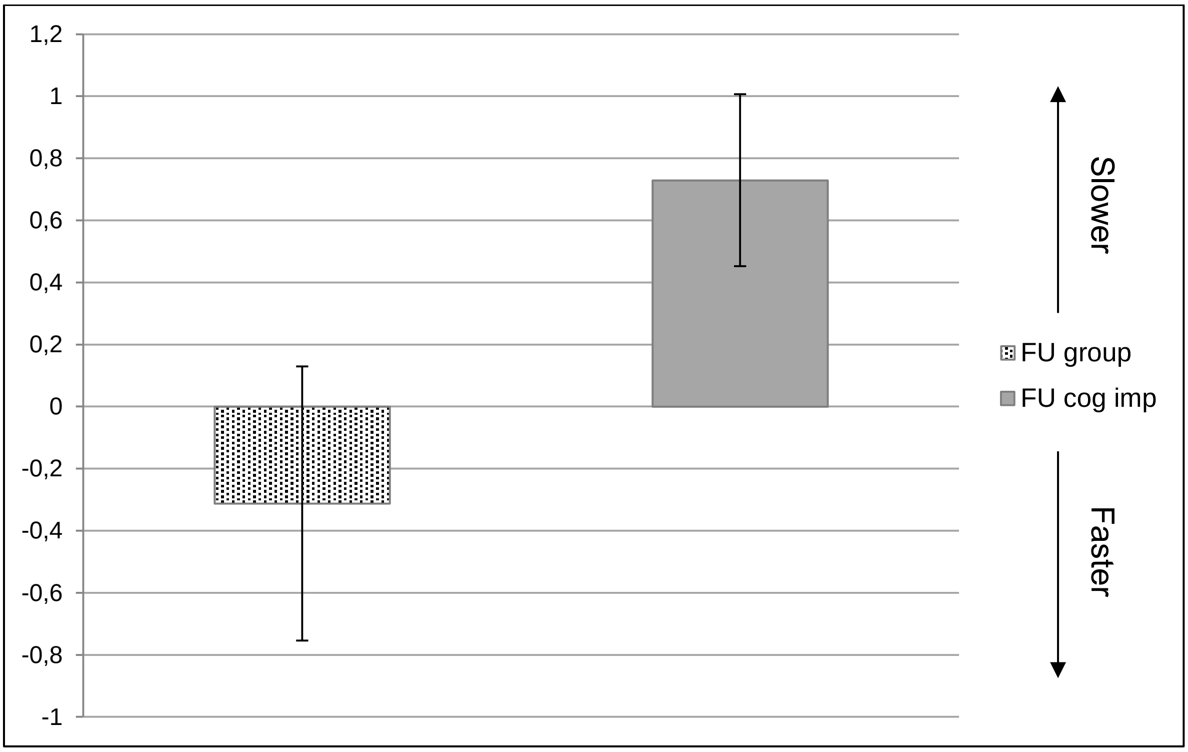

Supplementary Figure S1 Change of the results of the Trail Making Test B over 9 months

Differences of performance in seconds between study visit 1 and 3 were transformed using Johnson transformation; presentation of means and standard errors of transformed differences (negative values = faster performance; positive values = slower performance); follow-up group with suspected premorbid cognitive impairment (FU group cog imp; N=9) and a subgroup of patients without evidence of premorbid cognitive impairment matched to the other group in age, sex, and educational level (FU group; N=9)

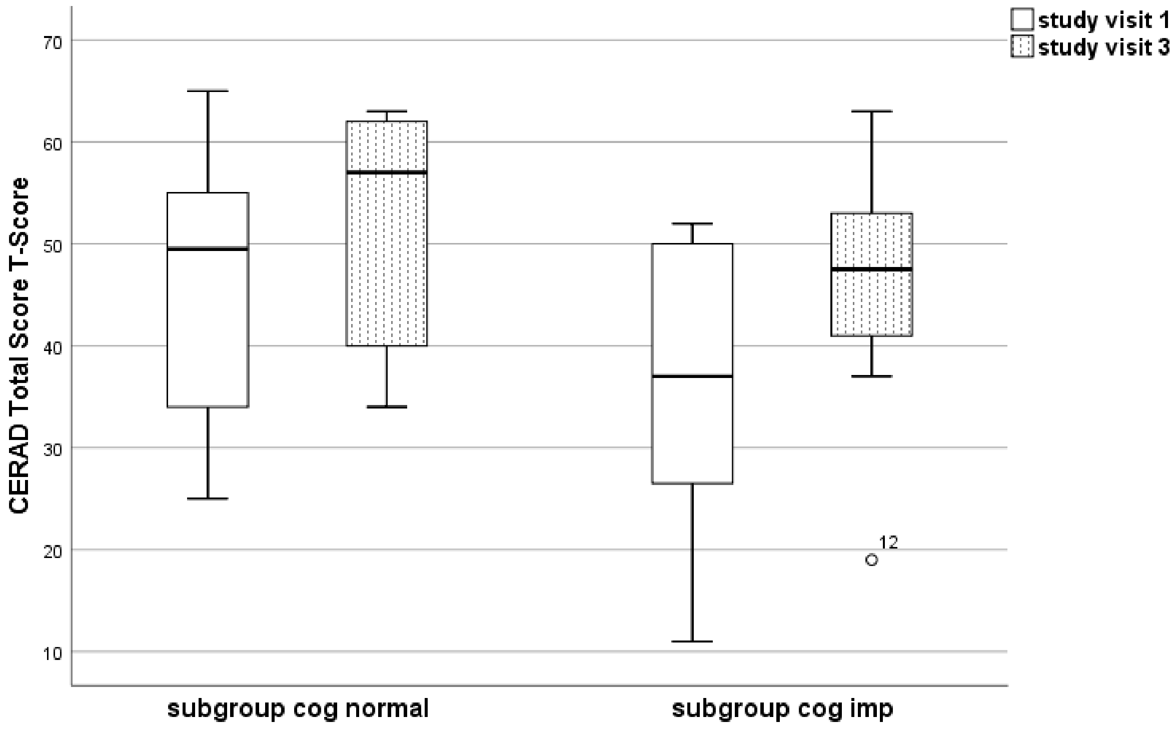

Supplementary Figure S2 Distribution of the T-Scores of the results of both matched subgroups without and with evidence of premorbid cognitive impairment at study visit 1 and 3

Distribution of the T-Scores (transformed values of the Consortium to Establish a Registry for Alzheimer´s Disease CERAD Total Score + demographical correction factor) of the both subgroups: subgroup without evidence of premorbid cognitive impairment (cog normal) and subgroup with evidence of premorbid cognitive impairment (cog imp). Only patients who underwent both study visits are considered, both groups are matched in age, sex and educational level. T-scores of normative data are characterized by mean=50 and SD=10; maximum value is 65, minimum value is 10; normative data are available for the CERAD Total Score of subjects between 50 and 90 years of age; subgroup cog normal: N=8; subgroup cog imp N=8

p-value: ** p<0.01

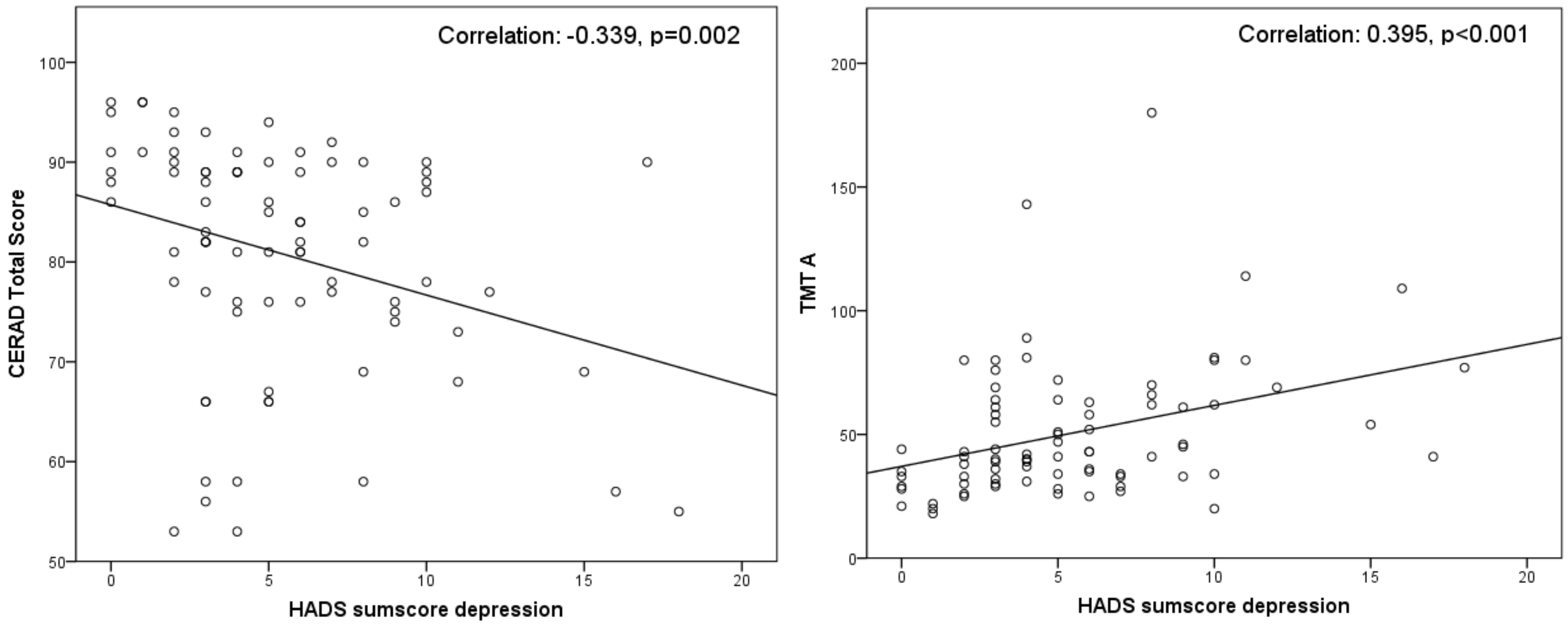

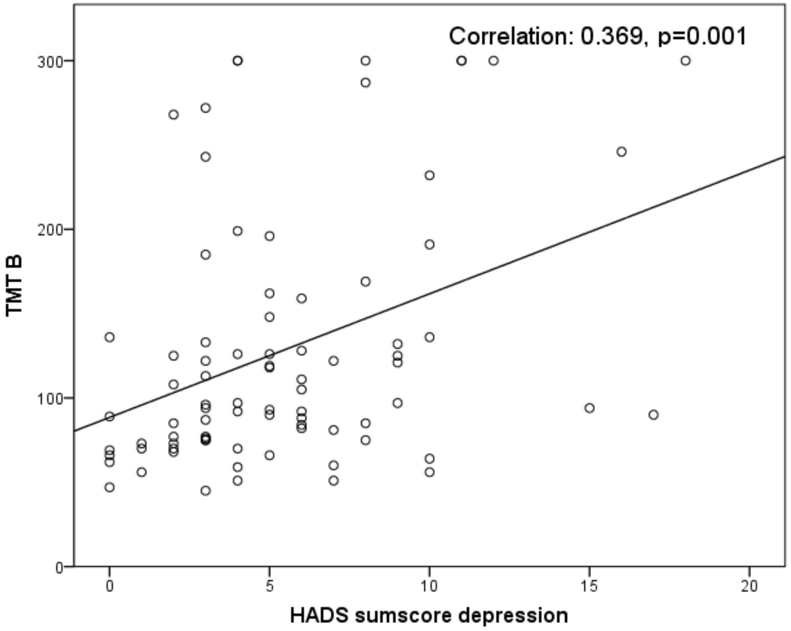

Supplementary Figure S3

Correlations between cognitive parameters and depression score

Cognitive parameters are: Consortium to Establish a Registry for Alzheimer´s Disease CERAD Total Score, Trail Making Tests (TMT) A and B; sumscore depression from the Hospitality Anxiety and Depression Scale (HADS), Spearman Rank correlation; correlation coefficients and p-values

Supplementary Text

Definition of selected covariates

Vascular Risk Score: To quantify the cardiovascular burden of the participants we used the risk score of Essen which is a validated tool in the clinical setting to estimate the probability of a new stroke.[1] It is composed by 8 items with maximum one point per item except for age that ranges from 0 to 2 points. We considered the medical history including the diagnosis at admission. The items of the score are:

- Age <65 years — 0
- Age 65-75 years — 1 point
- Age >75 years — 2 points
- Arterial Hypertension — 1 point
- Diabetes mellitus — 1 point
- Cardiac infarction in history — 1 point
- Other cardiovascular event — 1 point
- Peripheral arterial occlusive disease — 1 point
- Smoking — 1 point
- Stroke in history — 1 point

A score of more than 2 points is associated with a risk of ≥4% for a new stroke within one year.

Dosages of propofol, midazolam and sufentanil: With regard to past studies[2,3,4] we aimed to consider narcotics, benzodiazepines and opiates as potential toxic agents which may influence the cognitive outcome after ICU treatment. Due to the most frequent use on ICU propofol, midazolam and sufentanil were chosen as representative drugs of the three medication groups.

Charlson Comorbidity Index (CCI): The CCI is a score which predicts the ten-year mortality according to the comorbid conditions. The following conditions are considered: 1 point for each condition: myocardial infarction, congestive heart failure, peripheral vascular disease, dementia, cerebrovascular disease, chronic lung disease, connective tissue disease, ulcer, chronic liver disease and diabetes; 2 points: hemiplegia, moderate or severe kidney disease, diabetes with complication, tumor leukemia, lymphoma; 3 points: moderate or severe liver disease. 6 points are for malignant tumor, metastasis and AIDS. The CCI is one of the most frequent used scores in oncological departments. It ranges from 0 to 33 points, 7 points or more predict a 0% survival.[5]

Sequential Organ Failure Assessment Score (SOFA): This score is a widely-used scale on ICU to estimate the severity of illness over the time. It covers six different scores related to the organ systems: respiratory, central nervous, cardiovascular, hepatic, renal system and coagulation. Each score differs between 0 for no dysfunction and 4 for failure. The SOFA score is measured daily and provides information about the risk of mortality.[6]

References

1. Diener HC, Weber R. Akuttherapie und Prävention des ischämischen Schlaganfalls. *Journal für Neurologie Neurochirurgie und Psychiatrie* **12(1)**, 50-54 (2011).

2. Supplement to: Pandharipande PP *et al.* Long-term cognitive impairment after critical illness. *N Engl J Med*. **369**, 1306-16 (2013).

3. Jackson JC *et al*. Long-term cognitive and psychological outcomes in the awakening and breathing controlled trial. *Am J Respir Crit Care Med*. **182(2)**, 183-191 (2010).

4. Davydow DS, Zatzick D, Hough CL, Wayne JK. In-hospital acute stress symptoms are associated with impairment in cognition 1 year after intensive care unit admission. *Ann Am Thorac Soc*. **10**, 450–457 (2013).

5. Charlson ME, Pompei P, Ales KL, MacKenzie CR. A new method of classifying prognostic comorbidity in longitudinal studies: development and validation. *J Chronic Dis.* **40**, 373-83 (1987).

6. Ferreira FL, Bota DP, Bross A, Mélot C, Vincent JL. Serial evaluation of the SOFA score to predict outcome in critically ill patients. *JAMA* **286**, 1754-8 (2001).
